# The Importance of Bright Spotty Lesions on Magnetic Resonance Imaging in Predicting Chronic Neuropathic Pain in Myelitis

**DOI:** 10.3390/jcm13247820

**Published:** 2024-12-21

**Authors:** Je Hong Min, Sung-Yeon Sohn, In Soo Joo

**Affiliations:** Department of Neurology, School of Medicine, Ajou University Medical Center, Ajou University, Suwon 16499, Republic of Korea; 6jason9@naver.com (J.H.M.); sungyeonsohn@gmail.com (S.-Y.S.)

**Keywords:** chronic neuropathic pain, bright spotty lesions, prognostic factors, myelitis, corticosteroids

## Abstract

**Background/Objectives**: Chronic neuropathic pain (CNP) stands as one of the most debilitating complications in patients with myelitis owing to its challenging management. Bright spotty lesions (BSLs) are frequently observed in neuromyelitis optica spectrum disorder (NMOSD), but few reports have discussed CNP in myelitis. We aim to demonstrate that BSLs could be one of the potential prognostic factors for CNP development in myelitis. **Methods**: We examined 63 patients diagnosed with myelitis. Patients were categorized into CNP and non-CNP groups. We assessed the severity of clinical symptoms and the oral steroid dose administered after pulse therapy. Spine magnetic resonance imaging (MRI) of each patient was reviewed to analyze the characteristics of myelitis. Serological and cerebrospinal fluid (CSF) findings were also examined to confirm the etiology. **Results**: CNP was observed in 27 patients (42.9%). The mean onset age of patients with CNP was 45.26 ± 14.16 years. The MRI lesions exhibited more enhanced features and bright spotty lesions (BSLs) in the CNP group (χ^2^ test, *p* < 0.05). Patients with CNP received a lower oral steroid dose during the first month after symptom onset (χ^2^ test, *p* < 0.05). Multivariate logistic regression analysis revealed that patients with CNP exhibited significant BSLs in their myelitis lesions on spine MRI (OR 4.965; 95% CI, 1.282 to 19.235, *p* = 0.02). **Conclusions**: Although the exact mechanism remains unknown, the presence of BSLs on spine MRI could serve as an independent prognostic factor for CNP development. Additionally, our study suggests that lower oral steroid doses administered immediately after symptom onset are associated with CNP development. Further investigation with a larger cohort is warranted to validate our findings.

## 1. Introduction

Neuropathic pain is defined by the International Association Study of Pain (IASP) as pain caused by the lesions or diseases involving the related somatosensory system, and when the pain persists more than 3 months, it is defined as a chronic neuropathic pain (CNP) [1]. Management of CNP still remains a major challenge, so patients with CNP still have poor quality of life with multiple complications, such as joint contracture, muscle atrophy, pressure sores, and cardiovascular problems [2].

Bright spotty lesions (BSLs) are defined as hyperintense intramedullary lesions in the spinal cord that are as or more intense than the surrounding cerebrospinal fluid (CSF) on axial T2-weighted images [3]. Additionally, to differentiate BSLs from syringomyelia, BSLs should appear brighter than the CSF on axial T1-weighted images [4]. It is well known that BSLs are highly specific in neuromyelitis optica spectrum disorder (NMOSD), so it can differentiate from other causes of myelopathy such as multiple sclerosis (MS) and myelin oligodendrocyte glycoprotein-associated disease (MOGAD) [5,6]. Several studies has been reported about the predictors of neuropathic pain in various CNS demyelinating diseases, such as MS [7,8], NMOSD [9,10], and MOGAD [11]. Some studies have reported that neuropathic pain prevalence showed a positive correlation with lesion length and cervical and thoracic distribution in MS [8,12]. Other clinical studies have suggested age, sex, and extended thoracic lesions to be independent factors for the development of neuropathic pain in NMOSD [10]. To the best of our knowledge, predictors of chronic neuropathic pain in cases of pure myelitis have rarely been reported. One previous study revealed that early-age onset and non-idiopathic transverse myelitis patients can easily suffer from neuropathic pain [13]. Since pain can significantly impair the quality of life in patients with myelitis [11,14], it is important to identify predictive factors for progression to neuropathic pain, and preventing its development in advance is crucial.

Therefore, we aimed to investigate various prognostic factors that contribute to chronic neuropathic pain (CNP) in myelitis including idiopathic transverse myelitis (iTM), and as a result, we discovered that BSLs are an important factor in the development of CNP.

## 2. Materials and Methods

### 2.1. Study Design and Ethical Approval

This retrospective study was conducted at Ajou University Hospital, a tertiary referral center in Suwon, South Korea. This study was performed according to the Declaration of Helsinki and approved by our Institutional Review Board. The requirement of informed consent was waived due to the retrospective nature of this study.

### 2.2. Patient Selection

We conducted a retrospective review of the medical records and the MRI scans of patients diagnosed with myelitis who were seen at our university hospital between January 2006 and February 2020. The inclusion criteria were (1) age ≥ 18 years and (2) a clinical episode consistent with myelitis. The exclusion criteria were (1) MRI findings suggesting compressive or traumatic myelopathy, (2) evidence of demyelinating lesions on brain MRI, (3) symptom progression to nadir < 4 h, (4) patients’ difficulty in accurately expressing their pain profile due to concurrent neuropsychiatric disorders, such as dementia and depression, and (5) pain from other conditions (e.g., musculoskeletal pain, such as arthralgia or myalgia).

A total of 63 patients with abnormal intramedullary cord signal changes on spinal MRI were included in this study. Patients with CNP were categorized according to the criteria outlined by the IASP for ‘neuropathic pain’ [1], which involves pain persisting for more than 3 months.

### 2.3. Clinical Characteristics

We collected the following data: demographics (age and sex), time to first diagnosis of myelitis and initiation of treatment, presence of myelitis relapse, and the total dose of corticosteroids used in the first 1 month and 3 months after symptom onset. Neurological disability was measured using the Extended Disability Status Scale (EDSS) [15]. The etiology of myelitis was classified as idiopathic, CNS demyelinating diseases (MS and NMOSD), infections, or those associated with other autoimmune diseases. MS was diagnosed based on the 2017 McDonald criteria [16], while NMOSD was diagnosed according to the 2015 International Panel for NMO Diagnosis criteria [17]. MOGAD was excluded due to the unavailability of the MOG-IgG antibody testing equipment at our center, as detecting MOG-IgG antibodies is essential for the diagnosis according to the recently updated international MOGAD criteria [18]. If no specific cause was identified after a thorough evaluation, we diagnosed iTM according to the criteria established by the 2002 Transverse Myelitis Consortium Working Group [19].

Total oral steroid doses were measured at 1 and 3 months after symptom onset in patients who had previously received intravenous methylprednisolone pulse therapy (1 g daily for 5 consecutive days) without additional immunomodulatory treatments, such as plasmapheresis, intravenous immunoglobulin, or immunosuppressants.

Demographic features and clinical characteristics of patients with or without CNP are summarized in Table 1.

### 2.4. Laboratory Tests

Routine chemistry panel and complete blood counts (CBCs) with differential counts were obtained immediately upon admission to our hospital. We analyzed the neutrophil-to-lymphocyte ratio (NLR), as the NLR is well known to be associated with neurological disability in CNS demyelinating disorders [20,21,22]. Advanced serological evaluations were performed based on individual clinical manifestations and included tests for vitamin D, vitamin B12, folate, copper, lead, syphilis serology, human T-lymphotropic virus 1 (HTLV-1), human immunodeficiency virus (HIV) serology, rheumatoid factor, antinuclear antibody, anti-neutrophil cytoplasmic antibody, angiotensin-converting enzyme, paraneoplastic antibodies, *Toxocara canis* IgG, total IgE, *Dermatophagoides pteronyssimus*, and *Dermatophagoides farinae*, aquaporin-4 (AQP4) antibody IgG, and other viral and parainfectious etiologies. AQP4 IgG tests were performed using indirect immunofluorescence on a substrate of mouse cerebellum and midbrain (before 2017) and using a live-cell-based assay (EUROIMMUN, Lübeck, Germany) since 2017.

Most patients underwent CSF analysis. CSF variables included cell count with differential, protein, glucose, virology (if indicated), IgG index, and oligoclonal bands (OCBs).

### 2.5. MRI Parameter Analysis

Spine MRI (and brain MRI, if necessary) was performed at our hospital using a 1.5 T or 3 T scanner (GE SIGNA™ HDxt 1.5 T scanner or GE Discovery™ MR750w 3.0 T scanner, GE HealthCare, Chicago, IL, USA) for most patients. Patients who underwent their initial MRI at another hospital during the first attack had their images transferred to and registered on our PACS system. Although the MRI protocols were somewhat heterogenous, we included at least axial and sagittal T1- and T2-weighted as well as contrast-enhanced sequences to evaluate the MRI characteristics of myelitis. Only one study lacked the contrast-enhanced sequence.

**Figure 1:**
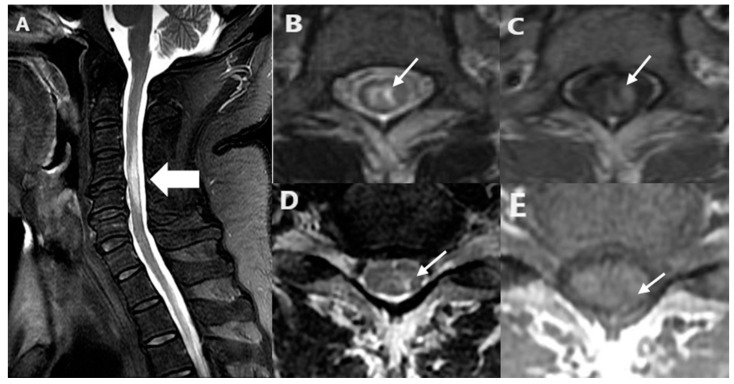
Spine MRI images of NTNC myelitis patients. (**A**) Sagittal T2-weighted images of cervical myelitis patient with sagittal BSLs (thick white arrow). Thoracic myelitis patient with centrally located BSLs (**B**,**C**) and cervical myelitis patients with peripherally located BSLs (**D**,**E**) on axial views. BSLs show very hyperintense lesions as high as the surrounding CSF on the T2-weighted image (thin white arrow) (**B**,**D**) and partially hypointense not as lowas the surrounding CSF on the T1-weighted image (thin white arrow) (**C**,**E**), which is different from permanent tissue loss.

Longitudinal extensive transverse myelitis (LETM) was defined as hyperintense lesions spanning more than 3 vertebral levels on sagittal T2-weighted images. BSLs were analyzed and categorized as centrally located, peripherally located, or both. Axial and sagittal T1-weighted contrast-enhanced images were assessed to determine the presence or absence of enhancement (Figure 1). Patients with demyelinating lesions on brain MRI were excluded from this study. Lesion location and BSLs were analyzed by two clinical neurologists blinded to the final diagnosis (J.H.M. and S.-Y.S.).

### 2.6. Statistical Analysis

Continuous data are presented as mean ± standard deviation (SD) or median (interquartile range [IQR], Q1–Q3), depending on the assumption of normal distribution. Student’s *t*-test and Mann–Whitney U test were employed accordingly for statistical analysis. Numeric variables were assessed for normality of data distribution using the Kolmogorov–Smirnov test. Categorial variables are described as percentages (%) and were analyzed using the chi-square test. A univariate logistic regression analysis was performed to identify potential predictors for the development of CNP. Multivariable logistic regression analysis was conducted with demographic factors and potential risk factors as covariates, selecting variables with a *p*-value of less than 0.1 in the univariable analysis. All statistical analyses were performed using IBM SPSS Statistics version 25.0 software.

## 3. Results

### 3.1. Demographic Characteristics of Patients

A total of 63 patients were included in this study; as shown in Table 1, 31 patients were women (49.2%), and 32 patients were men (50.8%). The mean age of the first attack was 44.5 ± 14.0 years. Patients without neuropathic pain or those with neuropathic pain persisting for less than 3 months were grouped as ‘without CNP’. Conversely, patients experiencing neuropathic pain persisting for 3 months or more were classified in the ‘CNP’ group.

Most patients underwent routine chemistry panel and CBC tests, but some patients had not undergone laboratory testing or visited our hospital without any medical records from other hospitals, resulting in a lack of serological findings. We reviewed specific laboratory parameters, when available, such as vitamin D levels and complement levels, as these are associated with the severity or development of neuropathic pain [23,24,25]. However, there was no significant difference in the serological profiles between the two groups. The albumin quotient (CSF albumin/serum albumin), an indicator of blood–brain barrier (BBB) breakdown [26,27,28], tended to be higher in CNP group but did not reach statistical significance (*p* = 0.198). The CSF data were also similar between the two groups.

The median time from first symptom onset to the diagnosis of myelitis was 3 weeks (interquartile range [IQR], 1–8 weeks). The age at symptom onset, sex differences and the etiology of myelitis did not differ between the two groups. Laboratory parameters in serum and CSF were also similar. The initial EDSS score from the first visit to our hospital did not show a significant difference, but the EDSS score 3 months after symptom onset tended to indicate a higher tendency for the CNP group (median 2.5, [IQR] 2.0–3.0, *p* = 0.038).

### 3.2. MRI Characteristics of Patients with Myelitis

In the spine MRI findings, one patient did not undergo contrast-enhancing imaging during the initial visit. While lesion level and length did not differ between the groups (median 3.0 segments, [IQR] 2.0–6.0, vs. 4.0 segments, [IQR] 4.0–8.0, *p* = 0.134), patients with CNP showed a high tendency for LETM (81.5%) and more centrally located lesions, although this did not reach statistical significance (*p* = 0.051, both). Additionally, there were higher ratios of BSLs and gadolinium enhancement on axial views in the CNP groups (*p* < 0.001 and *p* = 0.043, respectively).

### 3.3. Oral Steroid Doses After Intravenous Methylprednisolone Pulse Therapy

Patients with CNP took a lower dose of oral steroids during the first month after symptom start (*p* = 0.041), and this result remained consistent after adjusting for patients’ weight (average 11.75 mg/kg in non-CNP group vs. 6.27 mg/kg in CNP group, *p* = 0.019). However, there was no difference in the total oral steroid dose during the first 3 months after symptom onset (*p* = 0.613), even after weight adjustment.

### 3.4. Logistic Regression Analysis for Predictors of Chronic Neuropathic Pain

**Figure 2:**
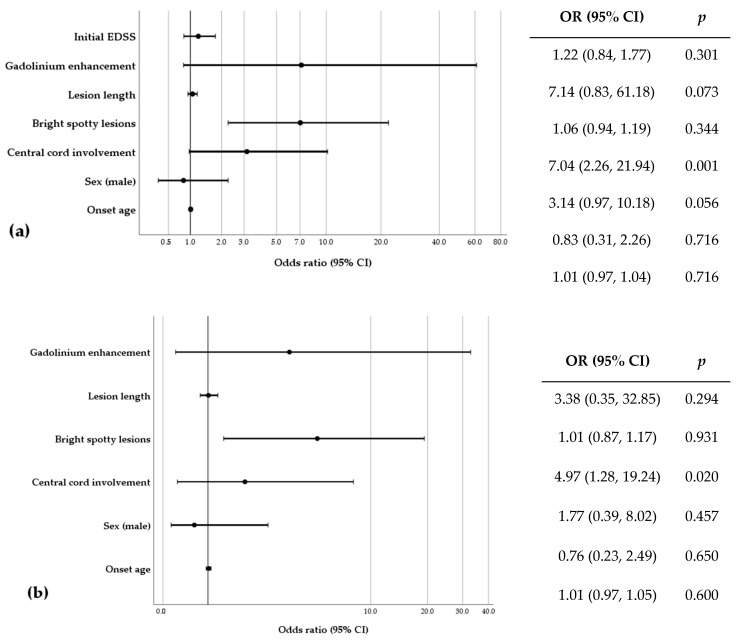
Forest plots of odds ratios from logistic regression analysis for the development to CNP in 62 patients with myelitis: (**a**) univariate and (**b**) multivariate analysis. Variables included in multivariate analysis are age, sex, central cord lesion, BSLs, lesion length, and gadolinium enhancement.

Univariate logistic regression was performed to evaluate potential predictors for the development to CNP. The following factors were included: (1) onset age, (2) sex, (3) central cord involvement on axial MRI views, (4) BSLs, (5) etiology (idiopathic or non-idiopathic), (6) myelitis lesion length, and (7) EDSS score at 3 months after symptom onset. Oral steroid dose for the first month after symptom onset showed a negative correlation with the occurrence of CNP (crude odds ratio [OR]: 0.998, 95% confidential interval [CI]: 0.997–1.000; not included in this report). However, this parameter was not included in the multivariate analysis due to the small sample size (n = 37). The presence of BSLs on spine MRI was the only predictive factor for the development of CNP in myelitis patients, and this result remained consistent after adjusting for onset age, sex difference, the presence of a central cord lesion, lesion length, and EDSS score after 3 months from symptom onset (Figure 2).

## 4. Discussion

In our study, we reviewed several potential predictors for CNP in myelitis patients. First, patients with CNP tended to have higher EDSS scores at 3 months after symptom onset. This finding suggests that neuropathic pain may influence neurological sequelae following a myelitis event. As Zhang et al. [29] reported that some functional scores, such as pyramidal or sensory domain scores, were higher in NMOSD patients with neuropathic pain, which could explain the above result. Moreover, although it was not statistically significant, patients with CNP tended to have longer lesions and a higher likelihood of LETM. Naturally, longer myelitis lesions could result in greater neurological disability (indicated by higher EDSS scores), and some studies have demonstrated these findings [30,31]. However, the initial EDSS score was not associated with development to CNP.

On spine MRI findings, we observed that more gadolinium-enhanced lesions were detected in patients with CNP after a myelitis attack. When interpreted in conjunction with the albumin quotient, the presence and degree of BBB disruption may be positively associated with the development of CNP. This is not surprising, as BBB breakdown indicates more severe inflammation and is associated with disease progression in CNS disorders [32]. The blood–spinal cord barrier (BSCB), located in the spinal cord, functions similarly to the BBB [33]. Gadolinium enhancement in the spinal cord also reflects BSCB disruption [34], which may contribute to the acceleration of myelitis progression, potentially resulting in functional sequelae such as CNP. However, we failed to demonstrate a statistically significant relationship between gadolinium enhancement and the development of CNP in the regression analysis (univariate OR 7.143 [0.834–61.178], *p* = 0.073).

The most important finding in our study, from the perspective of the MRI parameters, was that most myelitis patients with CNP exhibited BSLs on spine MRI (70.8%, *p* < 0.001). Several studies have demonstrated that BSLs have greater specificity in NMOSD compared to other demyelinating diseases [3,4,5,35]. In our study, although statistical significance was not observed, more than half of the patients diagnosed with NMOSD had myelitis with BSLs (Table 2). The correlation between BSLs and neuropathic pain seems reasonable, given that neuropathic pain is more severe in NMOSD than in other CNS demyelinating diseases, such as MS [36,37] and MOGAD [37].

While the pathophysiology of BSLs is not well understood, it is postulated that these lesions result from cystic inflammation due to the loss of AQP4 channels, which are located on the foot processes of astrocytes [5,38]. This could explain why central neuropathic pain is more severe in AQP4-positive myelitis patients [10] and why neuropathic pain is more common in NMOSD compared to other CNS demyelinating diseases [36,39]. Furthermore, BSLs are thought to reflect the severity of damage in the spinal cord, particularly in the gray matter, possibly involving the central canal [3]. Given that the mechanism of neuropathic pain is largely associated with the spinothalamic tract, which decussates through the gray matter of the spinal cord, BSLs may play a significant role in the generation of neuropathic pain in myelopathy.

In Table 2, our patients showed a slight tendency toward an association between AQP4 positivity and BSLs, but this did not reach statistical significance (11.1% vs. 26.7%, *p* = 0.268). However, patients with BSLs had longer myelitis lesions (6.0 segments, [IQR] 3.0–10.0, vs. 3.0 segments [IQR] 2.0–5.0, *p* = 0.003) and a greater tendency for centrally localized lesions compared to those without BSLs (95.8% vs. 51.3%, *p* < 0.001) with a high likelihood of gadolinium-enhanced lesions (100.0% vs. 76.3%, *p* = 0.010).

Another interesting finding of this study is that patients who received a relatively low dose of oral prednisone during the first month after the symptom onset more frequently suffered from CNP. It is well known that early immunotherapy for immune-mediated disorders can lead to better outcomes and fewer complications [40,41,42]. Akaishi et al. [43] demonstrated that early initiation of oral steroids can prevent disease relapse and reduce the severity of depression and fatigue in NMOSD patients. Similarly, Min et al. [44] recently reported that early steroid therapy may result in favorable visual acuity outcomes in the first episode of optic neuritis, particularly in cases associated with NMOSD or MOGAD.

In this context, our study also showed that the development of CNP appears to be associated with the dose of oral corticosteroids administered within the first month after symptom onset (687.81 ± 417.12 mg vs. 395.71 ± 414.05 mg, *p* = 0.041). This result remained consistent after adjusting for patients’ individual body weight (11.75 ± 7.31 mg/kg vs. 6.27 ± 6.20 mg/kg, *p* = 0.019). However, there was no significant difference in the total oral corticosteroid dose administered within the first 3 months after symptom onset between the CNP and non-CNP groups. Additionally, although not mentioned in this article, the time taken to taper oral corticosteroid from 1 mg/kg/day to 20 mg/day tended to be shorter in the CNP group than in the non-CNP group, but this did not reach statistical significance (n = 34, 25.40 ± 6.52 days vs. 31.71 ± 15.69 days, *p* = 0.115). This result may suggest that not only the dose of corticosteroid but also the tapering duration may be an important factor for the development of CNP in myelitis. A further large-sample cohort study will support this finding. These findings suggest that the early appropriate immunotherapy can prevent CNP in myelitis patients and can improve the quality of life after the diagnosis.

Our study has several limitations. First, due to the retrospective nature of this study, we were unable to assess the characteristics, locations, and severity of neuropathic pain. As a result, it was difficult to determine whether the pain was at-level or below-level neuropathic pain, which depends on the location of pain [45]. Additionally, we could not analyze the correlation with pain severity and potential predictive factors of neuropathic pain. Second, we analyzed patients who visited our clinic for the first time after a myelitis attack. This implies that patients with a history of previous myelitis attacks could have experienced worsened pain following a subsequent attack. Lastly, the sample size of the patients who received oral prednisone was small (n = 37). Furthermore, we did not include patients treated with a combination of corticosteroids and other immunotherapies in our analysis, as we did not consider potential synergistic effects. Therefore, further studies with more detailed evaluations and larger sample sizes are needed to strengthen our findings.

## 5. Conclusions

BSLs are commonly recognized as a key radiological factor in NMOSD. However, based on the proposed mechanisms of BSLs, they could serve as an independent predictive factor for the development of CNP in our myelitis patients. Additionally, the total oral steroid dose administered during the first month after symptom onset, assuming patients were treated with steroid pulse therapy, could also be an important factor influencing the risk of CNP. However, due to the small size of our research group, further large-cohort studies are needed to validate these findings.

## Figures and Tables

**Table 1:** Demographic, laboratory, and imaging profiles of myelitis patients (n = 63).

	Patients Without CNP (n = 36)	Patient with CNP (n = 27)	*p*
**Onset age (years)**	43.97 ± 14.06	45.26 ± 14.16	0.721
**Time to first diagnosis and treatment (weeks) (median, IQR)**	2.0 (1.0–8.0)	3.0 (1.0–5.0)	0.834
**Sex (male) (n, %)**	19 (59.4%)	13 (40.6%)	0.716
**Lesion level (n, %)**			0.510
Cervical	16 (64.0%)	9 (36.0%)	
Thoracic	9 (60.0%)	6 (40.0%)	
Lumbosacral	0 (0.0%)	0 (0.0%)	
Multi-level	11 (47.8%)	12 (52.2%)	
**Lesion length (segments) (median, IQR)**	3.0 (2.0–6.0)	4.0 (3.0–8.0)	0.134
**LETM (n, %)**	21 (58.3%)	22 (81.5%)	0.051
**Axial characteristics of spine MRI (n, %)**			
Bright spotty lesions	7 (29.2%)	17 (70.8%)	<0.001
Central cord involvement	21 (58.3%)	22 (81.5%)	0.051
Gadolinium enhancement (n = 62)	28 (77.8%)	25 (96.2%)	0.043
**Initial EDSS (median, IQR)**	2.5 (2.0–3.0)	3.0 (2.0–3.5)	0.207
**EDSS at 3 months (median, IQR)**	2.0 (1.0–2.5)	2.5 (2.0–3.0)	0.038
**Aquaporin-4 antibody (n = 59)**	4 (11.8%)	5 (20.0%)	0.385
**Laboratory findings**			
WBC (/μL) (n = 60)	7301.18 ± 2582.47 (n = 34)	7465.38 ± 2191.98 (n = 26)	0.796
ANC (/μL) (n = 59)	4513.91 ± 1737.12 (n = 33)	4554.70 ± 1737.12 (n = 26)	0.940
Lymphocyte (/μL) (n = 59)	1984.32 ± 766.83 (n = 33)	2155.33 ± 918.41 (n = 26)	0.439
NLR (n = 59)	2.66 ± 2.05 (n = 33)	2.43 ± 1.52 (n = 26)	0.627
Erythrocyte sedimentation rate (mm/h) (n = 59)	10.64 ± 9.89 (n = 33)	8.88 ± 6.23 (n = 26)	0.410
C-reactive protein (mg/dL) (n = 58)	0.20 ± 0.52 (n = 32)	0.09 ± 0.10 (n = 26)	0.302
25-hydroxy-vitamin D (ng/mL) (n = 47)	23.88 ± 14.22 (n = 27)	21.74 ± 10.00 (n = 20)	0.569
Albumin quotient (Qalb) (×10^−3^) (n = 52)	9.19 ± 11.0 (n = 29)	19.14 ± 34.73 (n = 23)	0.198
**CSF parameters**			
WBC (/μL) (n = 59)	18.94 ± 51.13 (n = 34)	8.12 ± 24.08 (n = 25)	0.331
Protein (mg/dL) (n = 59)	58.14 ± 50.49 (n = 34)	74.65 ± 69.36 (n = 25)	0.294
IgG index (n = 53)	0.76 ± 0.32 (n = 30)	0.60 ± 0.28 (n = 23)	0.064
**Total oral prednisone dose for 1 month from symptom onset (mg) (n = 37)**	687.81 ± 417.12 (n = 16)	395.71 ± 414.05 (n = 21)	0.041
**Total oral prednisone dose for 1 month from symptom onset, adjusted by body weight (mg/kg) (n = 37)**	11.75 ± 7.31 (n = 16)	6.27 ± 6.20 (n = 21)	0.019
**Total oral prednisone dose for 3 months from symptom onset (mg) (n = 37)**	1369.69 ± 589.74 (n = 16)	1502.86 ± 905.97 (n = 21)	0.613
**Total oral prednisone dose for 3 months from symptom onset, adjusted by body weight (mg/kg) (n = 37)**	23.84 ± 11.59 (n = 16)	22.95 ± 12.15 (n = 21)	0.823
**Recurrent of myelitis (n, %)**	13 (36.1%)	10 (37.0%)	0.940
**Etiology of myelitis (n, %)**			0.644
Idiopathic	20 (55.6%)	15 (55.6%)	
NMOSD	7 (19.4%)	8 (29.6%)	
MS	2 (5.6%)	0 (0.0%)	
Infection	2 (5.6%)	2 (7.4%)	
Connective tissue disease *	4 (11.1%)	1 (3.7%)	
Others **	1 (2.8%)	1 (3.7%)	

* Myelitis associated with mixed connective tissue diseases, such as systemic lupus erythromatosus and Behcet’s disease. ** One case of paraneoplastic myelopathy was suspected; the other case was diagnosed with adult polyglucosan body disease.

**Table 2:** Demographics and clinical characteristics in myelitis patients with or without BSLs (n = 63).

	Patients Without BSLs (n = 39)	Patient with BSLs (n = 24)	*p*
**Onset age (years)**	44.33 ± 15.31	44.83 ± 11.89	0.892
**Sex (male) (n, %)**	20 (51.3%)	12 (50%)	0.921
**Lesion level (n, %)**			0.174
Cervical	19 (48.7%)	6 (25.0%)	
Thoracic	8 (20.5%)	7 (29.2%)	
Lumbosacral	0 (0.0%)	0 (0.0%)	
Multi-level	12 (30.8%)	11 (45.8%)	
**Lesion length (segments) (median, IQR)**	3.0 (2.0–5.0)	6.0 (3.0–10.0)	0.003
**Axial characteristics of spine MRI (n, %)**			
Gadolinium enhancement (n = 62)	29 (76.3%)	24 (100.0%)	0.010
Central cord involvement	20 (51.3%)	23 (95.8%)	<0.001
**LETM**	21 (53.8%)	22 (91.7%)	0.002
**Recurrent of myelitis (n, %)**	13 (33.0%)	10 (41.7%)	0.505
**Albumin quotient (Qalb) (×10^−3^) (n = 52)**	14.97 ± 28.30	11.71 ± 19.32	0.644
**CSF parameters**			
WBC (/μL) (n = 59)	20.50 ± 52.04	4.74 ± 12.75	0.090
Protein (mg/dL) (n = 59)	67.40 ± 71.01	61.59 ± 34.72	0.717
IgG index (n = 53)	0.70 ± 0.33	0.66 ± 0.28	0.644
**Aquaporin-4 antibody (n = 59) (n, %)**	4 (11.1%)	5 (21.7%)	0.268
**Etiology of myelitis (n, %)**			0.419
Idiopathic	21 (53.8%)	14 (58.3%)	
NMOSD	7 (17.9%)	8 (33.3%)	
MS	2 (5.1%)	0 (0.0%)	
Infection	3 (7.7%)	1 (4.2%)	
Connective tissue disease	4 (10.3%)	1 (4.2%)	
Others	2 (5.1%)	0 (0.0%)	
**Total oral prednisone dose for 1 month from symptom onset (mg)**	597.9 ± 430.0 (n = 21)	422.5 ± 434.8 (n = 16)	0.229
**Total oral prednisone dose for 1 month from symptom onset, adjusted by body weight (mg/kg)**	10.14 ± 7.46 (n = 21)	6.67 ± 6.43 (n = 16)	0.146
**Total oral prednisone dose for 3 months from symptom onset (mg)**	1355.7 ± 589.5 (n = 21)	1540.3 ± 964.2 (n = 16)	0.476
**Total oral prednisone dose for 3 months from symptom onset, adjusted by body weight (mg/kg)**	23.11 ± 11.27 (n = 21)	23.62 ± 12.73 (n = 16)	0.898

## Data Availability

Data can be made available on request by the authors.
